# Variable Stiffness Structures in Biomimetic Robotic Fish: A Review of Mechanisms, Applications, and Challenges

**DOI:** 10.3390/biomimetics11030219

**Published:** 2026-03-18

**Authors:** Hua Shao, Cong Lin, Zhoukun Yang, Luanjiao Deng, Jinfeng Yang, Xianhong He, Fengran Xie

**Affiliations:** 1 The Key Laboratory of Metallurgical Equipment and Control Technology Ministry of Education, Wuhan University of Science and Technology, Wuhan 430081, China; shaohua@wust.edu.cn (H.S.); lc20030215@outlook.com (C.L.); atkun@wust.edu.cn (Z.Y.); 202403704219@wust.edu.cn (L.D.); 2 Institute of Applied Artificial Intelligence of the Guangdong-Hong Kong-Macao Greater Bay Area, Shenzhen Polytechnic University, Shenzhen 518055, China; 3 Undergraduate School of Artificial Intelligence, Shenzhen Polytechnic University, Shenzhen 518055, China; jfyang@szpu.edu.cn; 4 Library, Shenzhen Polytechnic University, Shenzhen 518055, China; bmhxh@szpu.edu.cn

**Keywords:** variable stiffness structures, biomimetic, robotic fish, smart materials, fluid-driven, hybrid-driven mechanisms

## Abstract

Biological fish possess the intrinsic ability to dynamically modulate body stiffness to adapt to varying fluid environments, thereby optimizing propulsive efficiency, swimming speed, and maneuverability. In contrast, this capability remains a significant challenge for most existing robotic fish, which typically rely on fixed-stiffness configurations. This article presents a comprehensive review of variable stiffness structures and their applications in biomimetic robotic fish. The associated technologies are systematically classified into four categories: smart material-driven, bio-inspired, fluid-driven, and hybrid-driven mechanisms. A comparative analysis of state-of-the-art prototypes is conducted, evaluating critical performance metrics including physical dimensions, maximum swimming speed, minimum turning radius, maximum turning rate, and Strouhal number. Furthermore, the specific advantages and technical limitations of each variable stiffness category are critically assessed. Finally, existing challenges in current research are identified, and prospective directions are proposed. The review demonstrates that variable stiffness technology offers significant potential to advance the hydrodynamic performance of robotic fish and facilitate their deployment in practical engineering applications.

## 1. Introduction

Compared with traditional propeller-driven Autonomous Underwater Vehicles (AUVs), bio-inspired robotic fish exhibit superior maneuverability, low noise [1,2,3] and strong concealment [4,5]. In recent years, the research and development of biomimetic robotic fish have gained growing attention from researchers [6,7,8]. Drawing inspiration from the efficient propulsion modes of fish evolved over millions of years [9] such robotic systems integrate interdisciplinary technologies including fluid mechanics, mechanical engineering, electronic technology, control theory, and computer science [10]. Extensive research has been conducted on their structural design [11,12,13] and motion control [14,15] and they show broad application prospects in military detection [16,17,18] marine monitoring [19,20,21] and aquatic organism observation [22,23].

Biological swimming modes are categorized into two types: Body and/or Caudal Fin (BCF) propulsion and Median and/or Paired Fin (MPF) propulsion [24]. The BCF propulsion mode has been studied for many years due to its excellent acceleration performance and high-speed performance [25,26,27,28] the MPF mode is more advantageous in maneuverability and stability. Different fish species adopt distinct propulsion modes. For instance, the BCF propulsion mode is better suited for high-speed cruising, while the MPF propulsion mode excels in gliding and long-distance locomotion [29]. Fish muscles act as critical flexible actuators, exerting a significant and intricate influence on body stiffness [30]. For anguilliform fishes, euthynnus alletteratus, and scomberomorus cavalla, muscle force serves to reduce passive bending stiffness [31,32]. During the swimming cycle, these species can modulate the mechanical properties of their bodies by adjusting the effective stiffness and damping of muscles in response to the timing of muscle activation [33]. In conclusion, natural fish are capable of optimize their body stiffness through muscle contractions to attain the most stable swimming speed [34].

Fish undulatory motion requires multiple degrees of freedom (DOFs) [35,36]. Traditional multi-joint and multi-link structures meet this demand but suffer from low energy efficiency and high costs. Thus, extensive research has adopted flexible tails with fixed stiffness for robotic fish to realize undulatory motion with minimal DOFs [37]. Benefiting from flexible components, robotic fish achieve excellent swimming speeds. Lu et al. [38] pointed out that the complexity of the deformation mechanism of flexible materials poses significant challenges to the analysis and optimization of robotic fish’s power cost, highlighting an urgent need for targeted theoretical models and optimization methods. To address this, they proposed an integrated scheme consisting of “dynamic modeling + power cost characteristic analysis + two-phase optimization framework”, which effectively solves the key problems of multi-flexible-joint biomimetic robotic fish, including difficulties in power cost analysis, low optimization efficiency, and the challenge of balancing locomotor performance with low energy consumption. Tian et al. [39] investigated the effects of length and stiffness of passively flapping flexible plates (mimicking caudal fins) attached to oscillating wave foils (mimicking fish bodies) on flow fields and propulsion performance. They concluded that a long caudal fin (l ≥ 0.2) combined with sufficient stiffness (KB ≥ 1.0) is key to enhancing propulsion performance. Zhang et al. [40] explored the coupling relationship between caudal stiffness and flapping frequency by simulating stiffness changes with ethylene-propylene rubber sheets of different shapes. They found that each flapping frequency corresponds to a unique “optimal stiffness” for maximum thrust, and thrust is more sensitive to stiffness changes at low frequencies. Wu et al. [41] pointed out that excessive flexibility of the caudal fin exerts a negative impact on propulsion, while moderate rigidity is found to enhance hydrodynamic performance. Lu et al. [42] adjusted stiffness by varying the thickness of spring steel, revealing a positive correlation between swimming frequency and optimal stiffness, verifying the importance of stiffness-frequency matching. He et al. [43] improved swimming stability through gradient stiffness fin rays. Deng et al. [44] validated that forward swimming and turning share the same optimal caudal fin stiffness using fins made of different materials.

As mentioned above, the optimal stiffness of flexible structures is usually determined experimentally and cannot be adjusted online (referred to as offline stiffness tuning), which limits performance improvement across diverse scenarios [45]. The most common method for offline stiffness tuning is replacing components such as elastic parts with different stiffnesses. Offline stiffness tuning prevents robotic fish from adjusting stiffness during swimming to achieve real-time optimal performance. Therefore, online stiffness tuning is a better choice for real-time performance improvement [46]. Inspired by fish’ s ability to dynamically adjust stiffness [47], online stiffness tuning can realign stiffness with operating conditions in real time. Its implementation mainly includes smart materials, bio-inspired mechanisms, and fluid-driven and hybrid-driven. However, the majority of existing design schemes for variable-stiffness robotic fish lack an integrated sensing-control closed-loop system [48,49] failing to achieve real-time and dynamic stiffness adjustment in response to actual operating conditions. To break through these limitations, researchers have conducted in-depth exploratory studies from multiple perspectives: Zolfagharian et al. [50,51,52] verified the remarkable performance of tunable bending models and composite foam metamaterial springs, which can effectively enhance the performance and robustness of stiffness modulation systems in dynamic marine environments. Meanwhile, the industry is driving the evolution of closed-loop morphological intelligence for robotic fish, which integrates strain sensing [53,54,55] and flow field sensing [56,57,58,59] technologies, and combines algorithms such as the Central Pattern Generator (CPG) and deep reinforcement learning [54,60,61] to realize autonomous matching between stiffness and dynamic swimming conditions. In terms of extreme environment adaptability, to improve the adaptability of stiffness regulation for underwater robots in extreme environments, researchers have proposed bionic structural optimization and material substitution strategies [62,63,64]. Non-metallic flexible structures have shown enormous potential in this field; for instance, the dielectric elastomer actuator (DEA) system developed by Li et al. [65] has been successfully applied in the Mariana Trench environment.

To swim faster, fish increase driving frequency and body stiffness to gain greater power [66]. Quinn et al. [46] pointed out that the most effective caudal fin stiffness increases with driving frequency, thus active control of the stiffness of flexible caudal fin robotic fish is imperative. Achieving rapid, large-range fish-like stiffness adjustment online is crucial for improving the performance and practical application of robotic fish, as there is no universal “optimal stiffness” that consistently ensures optimal swimming performance across different motion states. Tang et al. [67] confirmed that caudal fin stiffness is also a key factor affecting swimming performance optimization, considering the stiffness-tunable deformation effect of caudal fins in water. Qiu et al. [68] noted that stiffness adjustment rules significantly improve performance. Xu et al. [30] demonstrated that fish bodies with moderate stiffness not only achieve the highest propulsion efficiency but also exhibit deformation characteristics that most closely align with those of naturally swimming fish.

Physiological studies show that fish adjust body stiffness through the antagonism of axial muscles, raising two core questions [69]. The first is why adjust stiffness: there is no universal “optimal stiffness” for all needs, and the optimal stiffness varies with external inputs [46]. The second is whether the cost is worthwhile: muscle antagonism obviously produces negative work, requiring additional energy. The energy saved from improved efficiency may offset the energy loss caused by negative work. Liu et al. [69] for the first time incorporated the energy consumption of stiffness tuning into the total cost of transport, confirming that the energy saved by tunable stiffness can compensate for the energy consumption of the Tunable Stiffness Caudal Peduncle (TSCP) itself, validating the necessity of stiffness-tunable technology.

The body bending stiffness distribution (k) of fish is jointly influenced by structure, materials, and muscle regulation. Some natural fish exhibit non-uniform stiffness distribution along the body length. Most adult fish have a non-uniform distribution (higher in the anterior and lower in the posterior) along the anteroposterior axis, which enhances thrust and energy efficiency in inertial flow regimes (Re ≥ 2000). However, the advantages of non-uniform stiffness in intermediate flow regimes (1 ≤ Re ≤ 2000) remain unclear: Wang et al. [70] found that uniform stiffness optimizes the wake field at high frequencies, while Chen et al. [71] confirmed that both uniform and non-uniform distributions can achieve optimal performance, with non-uniform distribution more likely to achieve high speeds at low frequencies.

Recent studies on bistable mechanisms [72,73,74] have verified their favorable efficacy in programmable and adaptive stiffness regulation [75]. Mohammadi et al. [76] developed 3D-printed bistable mechanisms for tremor attenuation in wearable devices, which exhibit excellent vibration damping performance and customizable stiffness response characteristics. In contrast, Zolfagharian et al. [77] integrated such mechanisms into tuned mass damper (TMD) systems for vibration control, achieving adaptation to dynamic frequency changes through a passive solution and thereby enhancing the flexibility of traditional vibration control methods. Both studies underscore the application value of bistable mechanisms in engineering systems requiring adaptive vibration and stiffness modulation. Meanwhile, significant progress has been made in the design and modeling of adaptive flexible structures with stiffness switching capabilities. Mohammadi et al. [78,79] established an optimization framework integrating physics-enhanced neural networks (PENN) and reduced-order models (ROM), which enables the design of flexible structures based on desired stiffness profiles. By combining genetic algorithms and machine learning, this framework facilitates the rapid inverse development of mechanical metamaterials for variable-stiffness underwater robots, allowing the creation of adaptive structures with nonlinear stiffness behaviors such as buckling—thus meeting the application requirements of dynamic underwater environments.

In recent years, research on robotic fish with variable stiffness structures is more and more extensive, a comprehensive review of tunable stiffness structures for robotic fish is necessary. Previous studies have explored various aspects of this field: Ma et al. [80] summarized the research progress of smart material-based robotic fish, Quinn et al. [46] analyzed the mechanisms and advantages of tunable stiffness robotic fish, Xie et al. [81] reviewed the design of caudal fin-propelled robotic fish, Yan et al. [82] summarized the design, sensing, and autonomy of bio-inspired robotic fish, presenting eight typical branches.

**Figure 1:**
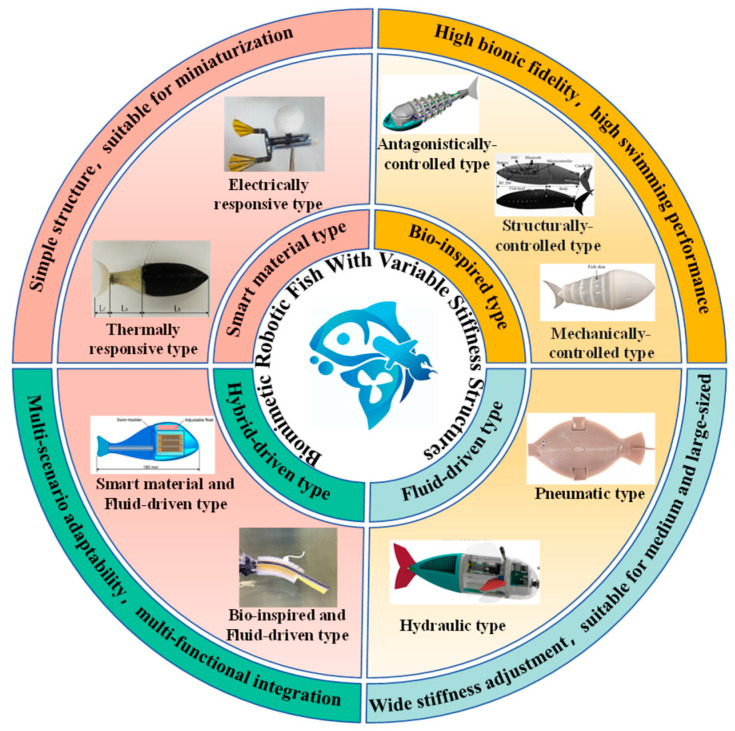
Applications of different variable-stiffness structures in biomimetic robotic fish. This figure classifies variable-stiffness structures into four major categories, while outlining their general application fields and key characteristics.

As depicted in Figure 1, this study provides a systematic review of variable-stiffness technologies in biomimetic robotic fish. While previous surveys, such as Quinn et al. [46], were restricted to bio-inspired structures, this work extends the scope to cover a broader spectrum of stiffness-tuning methodologies, including those utilizing smart materials, fluid driven, bio-inspired designs, and hybrid driven.

The paper is organized as follows. Section 2 presents variable-stiffness structures based on smart materials. Section 3 details bio-inspired stiffness modulation mechanisms, followed by Section 4, which elaborates on fluid-driven configurations. Hybrid-driven approaches are addressed in Section 5. Section 6 offers a comparative analysis of the swimming performance, summarizing the advantages, drawbacks and application scenarios of each category. Finally, Section 7 concludes the work, highlighting key challenges and proposing future development paths to support the evolution of this technology.

## 2. Smart Material Based Variable Stiffness Structure

### 2.1. Thermally Responsive Variable Stiffness Structure

**Figure 2:**
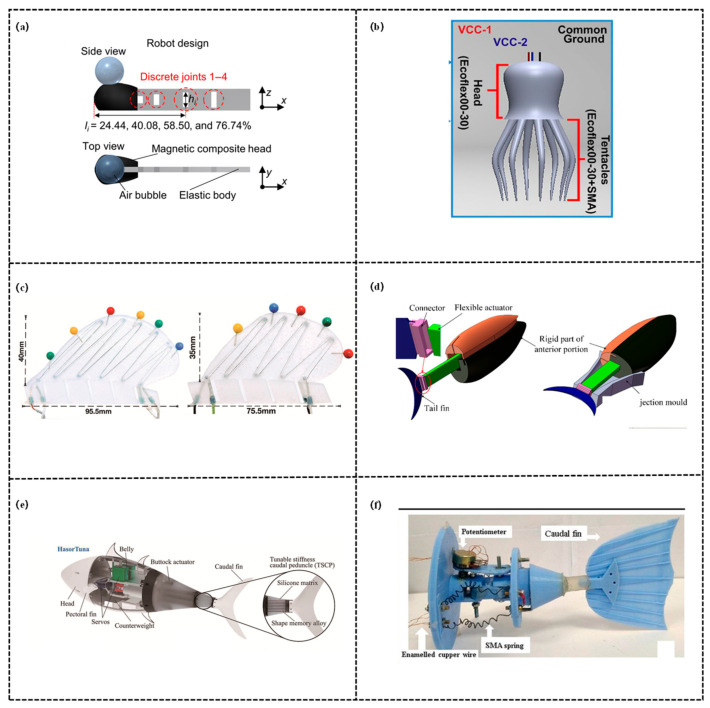
Application of thermally responsive variable stiffness structure in robotic fish. (**a**) Larval fish–like undulatory soft milliswimmers [70]. (**b**) Multi-material bio-inspired soft octopus robot (Octobot) [83]. (**c**) Robotic fish with soft dorsal/anal fin pairs [84]. Spherical reflective markers with different colors serve primarily as visual tracking identifiers for motion capture via high-speed cameras, bearing no additional classificatory implications. They are exclusively used to distinguish distinct monitoring positions on the fins, thereby enabling the 3D reconstruction of fin bending motions and the analysis of relevant kinematic parameters. (**d**) A soft robotic fish actuated by artificial muscle modules (SoRoFAAM 1) [85]. (**e**) Robotic fish with a tunable stiffness caudal peduncle (TSCP) [69]. (**f**) SMA-based carangiform robotic fish [86].

Thermally responsive variable stiffness structures are intelligent structures that achieve reversible stiffness switching by triggering internal microstructural rearrangement of materials via changes in ambient temperature, with their core relying on the thermally induced properties of Shape Memory Polymers (SMPs) and Shape Memory Alloys (SMAs), as shown in Figure 2.

Both types of materials can regulate Young’s modulus through temperature control, yet they exhibit differences in their working mechanisms and application scenarios: SMPs realize stiffness switching by virtue of the movement of polymer segments, rendering them suitable for low-stiffness and large-deformation scenarios, Wang et al. [70] designed a larval fish-like robot that mimics the morphology of larval zebrafish. This robotic fish is thermally responsive via SMPs: it innovatively integrates SMPs at its joints, leveraging the property of sharp temperature-induced variations in the elastic modulus of SMPs. Through water temperature regulation (27 °C/55 °C), the robot achieves stiffness distribution switching—exhibiting uniform stiffness at 27 °C and non-uniform stiffness at 55 °C. This water temperature-driven stiffness switching addresses the limitation of fixed stiffness in conventional robots.

In contrast, SMAs achieve a significant leap in stiffness via martensite-austenite phase transition, possess both high strength and self-recovery capabilities, and are more compatible with the propulsion-stiffness coordination requirements of biomimetic robotic fish. Ahmed et al. [83] developed a multi-material bio-inspired soft octopus robot (Octobot), which takes real octopuses as the bionic prototype and achieves stiffness regulation through the coordinated thermo-induced phase transition of SMAs. When energized, the SMAs are heated to 70 °C, transforming from the martensitic phase (low stiffness) to the austenitic phase (high stiffness) with contraction, after de-energization and cooling, they revert to the low-stiffness state. Coordinated with the asynchronous operation of SMA-α (main actuation) and SMA-β (antagonistic recovery), the robot dynamically adapts to the stiffness requirements during tentacle oscillation. Despite facing challenges including difficulty in SMA temperature control and unclear endurance performance, it provides a highly adaptable solution for underwater monitoring and promotes the advancement of bio-inspired soft robotics technology. Coral [84] focused on the limitation in 3D maneuverability of bio-inspired underwater robots, proposing a design of actuated fins mimicking the soft dorsal/anal fins of largemouth bass (*Micropterus salmoides*) to address the issue that most robotic fish are confined to horizontal propulsion and turning. The stiffness-tunable mechanism is achieved through the synergy between SMAs and silicone rubber. The core of the study lies in the development of lightweight, compact, and waterproof soft actuated fins: taking the fins of largemouth bass as the prototype, SMAs are embedded in silicone rubber without the need for external actuators, SMA wires on both sides are arranged in an antagonistic configuration, which contract upon energization to drive fin bending, with silicone rubber acting as a return spring. Additionally, 3D-printed “brackets” are used to fix the SMA wires and prevent slipping, enabling bidirectional bending of the fins. Tsimbo Fokou et al. [85] proposed a soft robotic fish, SoRoFAAM-1, actuated by SMA-based artificial muscle modules, aiming to enhance the bionic fidelity and locomotor performance of BCF (Body and/or Caudal Fin) propulsion-based soft robotic fish. The heating and cooling of SMAs are regulated via the Adaptive Regulated (AR) and Round-Robin heating strategies, enabling their transition between the martensitic phase (low stiffness) and austenitic phase (high stiffness). In the actuator, heated SMA wires contract to drive bending, while unheated SMA wires and spring steel provide a restoring moment, which dynamically adjusts the overall stiffness and enables precise bending and reciprocating motion. Liu et al. [69] focused their research on the tunable stiffness technology of fish-inspired robots, aiming to address the core question of whether the energy saved by tunable stiffness can offset the energy consumption of the stiffness-tuning mechanism itself. The research team developed a robotic fish named HasorTuna, innovatively designing a Tunable Stiffness Caudal Peduncle (TSCP) integrated with SMA. During swimming, the SMA wires are heated to harden, enabling the robotic fish to achieve both untethered swimming and online stiffness tuning. In offline tuning experiments, the researchers found that the stiffness of the caudal peduncle has a significant impact on swimming performance. In online tuning experiments, it was observed that the TSCP operates without additional energy consumption at high frequencies. For the first time, this study incorporated the energy consumption of stiffness tuning into the total cost of transport (COT), confirming that the energy saved by tunable stiffness can compensate for the energy consumption of the TSCP itself. This validates the necessity of tunable stiffness technology and provides a novel solution for the performance optimization of robotic fish. Koiri et al. [86] published a study focusing on the design of a carangiform-type robotic fishtail based on Shape Memory Alloys (SMAs), aiming to enhance forward thrust. Nitinol SMA springs were adopted as actuators, with phase transition triggered by regulating temperature via electric current—when energized and heated, the SMAs exhibit high shear modulus and stiffness in the hot state, generating contractile force, when de-energized and cooled, they possess low shear modulus and stiffness in the cold state. Cooperating with the antagonistic force of the origami-based prismatic frustum-type flexible skin to achieve reset, the stiffness is dynamically switched to drive the oscillation of the robotic fishtail.

Thermally responsive variable stiffness structures have emerged as a pivotal solution for stiffness regulation in miniature biomimetic robotic fish, owing to their stable material properties and compact structural configurations. Nevertheless, the complexity of temperature control and inherent slow response characteristics render them incompatible with application scenarios requiring high-frequency maneuverability of robotic fish.

### 2.2. Electrically Responsive Variable Stiffness Structure

**Figure 3:**
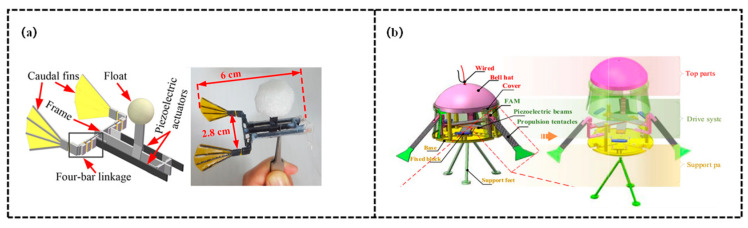
Application of electrically responsive variable stiffness structure in robotic fish. (**a**) Piezoelectric-driven bio-inspired micro-robotic fish with dual caudal fins [87]. (**b**) Bionic piezoelectric robotic jellyfish with a large deformation flexure hinge [88]. Figure 3b presents the complementary structural expression forms of the assembly schematic and exploded view of the bionic piezoelectric robotic jellyfish. Specifically, colors are uniquely coded by functional modules: blue-purple, red-orange and green tones identify the core innovative flexible amplification mechanism, the piezoelectric beam actuator as the power source and the propulsion tentacles as the execution unit, respectively; gray-white tones represent the auxiliary supporting and shell structures, while black and dark gray mark the connection and fixing positions, with the color saturation varying gradiently according to the component importance. A unified closed solid black frame is adopted to define the view scope and clarify the view type as a structural configuration diagram. In terms of line types, bold solid lines, thin solid lines and dashed lines are used to express the main contours of components, connection details and hidden assembly structures, respectively, and light-toned thin solid lines are additionally applied to distinguish the split components within the same module.

Electrically responsive variable stiffness structures achieve precise regulation of stiffness by triggering the reconstruction of mechanical properties of materials or systems via electrical signals. Lead Zirconate Titanate (PZT) enables bidirectional conversion of force, electricity, and deformation by virtue of its prominent piezoelectric effect, as shown in Figure 3.

The essence of its “stiffness tuning” lies in regulating the equivalent stiffness of the system through voltage to match the mechanical requirements of different motion scenarios. Possessing advantages of fast response speed, high energy density, and moderate driving voltage, PZT serves as a core driving component for miniature biomimetic robots. It can amplify micro-deformations via flexible hinges, transmission mechanisms, and other components, thereby realizing synergistic optimization of stiffness and propulsion. Zhao et al. [87] proposed a fast-moving piezoelectric micro bio-inspired fish to address the challenge of matching high maneuverability with low power consumption and low driving voltage for micro robotic fish. Stiffness regulation is achieved by adopting a “flexible hinge and transmission mechanism” scheme: polyimide (PI) flexible hinges provide basic flexibility while maintaining stable intrinsic stiffness, through the regulation of driving force via the piezoelectric effect of PZT and the deformation amplification by the four-bar linkage, the system dynamically adapts to the stiffness requirements during the oscillation of the dual caudal fins, balancing propulsion force and motion flexibility. This bio-inspired fish features a symmetrical dual-caudal-fin design, with its core actuation and transmission system composed of PZT bimorph cantilevers, rigid carbon fiber/resin composite structures, and PI flexible hinges. The four-bar linkage transmission mechanism amplifies the small deformation of the PZT cantilevers to drive the symmetrical oscillation of the dual caudal fins, the antagonistic forces generated by the caudal fins counteract lateral forces, thereby enhancing swimming stability and propulsion efficiency. Notably, the PZT-driven micro fish significantly outperforms similar micro fish actuated by IPMCs, SMAs, and DEs. Meanwhile, a theoretical model of kinematics and dynamics was established, which shows high consistency with experimental results. This model can be used to guide the design of piezoelectric-actuated caudal-fin robotic fish. Furthermore, the fish achieves a minimum turning radius of 1.4 cm, making it suitable for operations in narrow spaces. Xing et al. [88] addressed the limitation of small output displacement of piezoelectric ceramics (PZT) by developing a bionic piezoelectric robotic jellyfish with three tentacles and large-deformation flexure hinges, aiming to enhance the locomotor performance of underwater bionic robots. The driving system of this robotic jellyfish consists of a PZT beam, a flexible amplification mechanism (FAM), and carbon fiber tentacles. While the stiffness of PZT itself remains stable, system-level stiffness tuning is achieved through the combination of “voltage regulation and flexure hinge topology”. When a 100 V sinusoidal voltage is applied, the PZT generates bending deformation via the inverse piezoelectric effect, the parallel, triangular, and cross-topologies of the fishing line embedded in the FAM preset different baseline stiffness and amplify the small deformation of the PZT. By adjusting the driving frequency to match swimming requirements, the equivalent stiffness of the system is dynamically altered, ensuring that the swing amplitude of the tentacles matches the thrust force.

Electrically responsive variable stiffness structures have fulfilled the stiffness regulation requirements of miniature biomimetic robotic fish for narrow-space operations, courtesy of their advantages of fast response and high efficiency. However, their inherent limitations in output displacement and stiffness adjustment range render them incompatible with large-load and wide-range stiffness regulation scenarios for medium-to-large-sized robotic fish.

## 3. Bio-Inspired Variable Stiffness Structure

Bio-inspired variable-stiffness structures, drawing inspiration from the stiffness regulation mechanisms of natural organisms (e.g., fish muscle antagonism, fin morphological reconstruction, etc.), achieve dynamic stiffness adaptation aligned with biological motion characteristics through the synergy of structural design and control strategies.

### 3.1. Antagonistically-Controlled Variable Stiffness Structure

**Figure 4:**
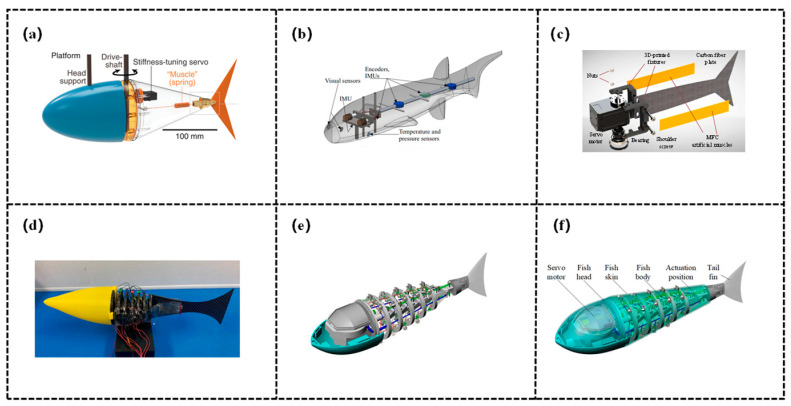
Application of antagonistic-controlled variable stiffness structure in robotic fish. (**a**) Tuna-inspired robotic fish [89]. (**b**) An electromyography-based robotic fish [90]. (**c**) Flexible fishtail with MFC. 3D-printed fixtures are used to connect the carbon fiber plate and the servo motor, while macrofiber composite (MFC) is attached to both sides of the carbon fiber plate [91]. (**d**) Robotic fish with DEA. The multi-module fish-inspired robot driven by fish-inspired white muscle-mimetic tapered dielectric elastomer actuator (DEA) modules [92]. (**e**) The tensegrity robotic fish (TenFiBot) [71]. The red lines in the figure represent the tension elements, which establish a symmetric horizontal tension network to achieve suspended support for the low-friction rotational joint and ensure the stability of the basic structure; the blue lines denote the actuation cables, acting as power transmission carriers that convert the rotational motion of the servo motor into linear tensile force; the green lines correspond to the MACCEPA springs, serving as the core of stiffness adjustment. Based on the principle of the prestressed singular elastic system, these springs enable a 14-fold range of stiffness regulation by increasing/decreasing their quantity or adjusting the pretension. (**f**) TenFiBot incorporating soft fish skin and tail flexibility [93].

Antagonistically-controlled variable stiffness structures achieve stiffness enhancement by applying two sets of forces in opposite directions to the same structure or joint (antagonistic co-contraction): it enhances stiffness by boosting the structure’s deformation resistance, conversely, adjusting the balance of these opposing forces reduces stiffness. This mechanism perfectly mimics the antagonistic regulation of axial muscles in fish, possessing both high biomimetic fidelity and precise controllability, as shown in Figure 4.

The variable stiffness realization via muscle/tendon antagonism mimics the antagonistic coordination of biological muscles and tendons, regulating stiffness through the bidirectional forces generated by cable/tendon-like structures or muscle-mimetic actuators. Zhong et al. [89] focused on the tunable stiffness technology and swimming performance optimization of fish-like robots and investigated how real fish leverage tunable stiffness—how they utilize muscular flexibility—to achieve efficient swimming by adjusting stiffness via their muscles. The robotic fish simulates the lateral tendon tension adjustment mechanism of tuna: it drives a muscle-mimetic spring using a motor, which pulls a polyethylene cable and a linear spring to alter the tension on the caudal fin connector. This, in turn, enables real-time adjustment of the torsional stiffness of the tail joint, thereby achieving active adjustability of stiffness. The swimming efficiency of the robot is maximized when the muscle tension is proportional to the square of its swimming speed, this strategy can double the efficiency of the tuna-like robot under operating conditions of 0–6 Hz and 0–2 BL/s, with a more pronounced energy-saving effect observed under high-frequency conditions. Furthermore, the tunable stiffness design can accommodate multi-speed and multi-task scenarios, providing a critical solution for the efficient operation of high-frequency fish-like robots. Martínez et al. [90] designed and developed an electromyography-based robotic fish. This robotic fish features a multi-link caudal spine connected in series with helical springs, integrated with an antagonistic tendon elastic system, and regulates stiffness based on Hill’s muscle model. The force from the electromagnetic oscillator is transmitted to the spring joints, by matching the spring stiffness coefficient with the moment of inertia and adjusting the torque through the feedback control law, it achieves adaptive stiffness of caudal vertebrae oscillation, thus adapting to the requirements of different swimming behaviors. Gu et al. [91] addressed the issue that existing robotic fish relied on passive flexibility or stiffness-tunable mechanisms but lacked dynamic adjustment capabilities, proposing a flexible fishtail design with muscle-like actuation. Specifically, this robotic fish adopted a servo motor as the primary driving joint and indirectly achieved dynamic stiffness adjustment by regulating caudal fin deformation via MFC artificial muscles attached to both sides. Furthermore, a partial differential equation (PDE) full-state observer was designed to perceive deformation, and a lightweight control framework was developed by integrating deep reinforcement learning (DRL), which was further deployed on an embedded platform. Experimental validations demonstrated that the fishtail’s thrust increased by 15% to 203% at frequencies ranging from 0.5 to 3 Hz, when integrated into an untethered robotic fish prototype, the swimming speed increased by up to 37% or decreased by up to 42% compared to the passive flexibility state, providing novel insights for the dynamic performance optimization of flexible robotic fish. Hitomi et al. [92] addressed the issues that the previously proposed fish-inspired white muscle-mimetic tapered dielectric elastomer actuator (DEA) modules lacked mathematical models and were difficult to integrate, completing the modeling of the modules and the development of a multi-module fish-inspired robot. Its stiffness-tuning mechanism is as follows: each module incorporates two antagonistic tapered DEAs, the energized DEA reduces the elastic restoring force due to Maxwell stress, breaking the left-right balance to achieve bending and reverting upon de-energization. Meanwhile, through the differentiated design of module parameters, the torque along the body axis is incrementally increased, indirectly adapting to the stiffness requirements of different body parts and ensuring swimming performance.

Elastic component antagonism employs springs, elastomers, and other elastic elements as core antagonistic components, achieving stiffness regulation through the interaction of elastic forces by adjusting the preload, deformation state, or combination mode of these elastic elements. Chen et al. [71] focused on the body stiffness variation of the tensegrity robotic fish, and proposed a stiffness-tuning method based on antagonistic stiffness in a kinematically singular configuration. The core of the study lies in the development of VSTJs, whose stiffness-tuning principle relies on antagonistic stiffness under a kinematically singular configuration: by integrating MACCEPA springs with tensegrity joints, the pretension of the springs is adjusted to further alter the torsional stiffness of the joints, thereby achieving controllable overall stiffness. The study designed TenFiBot and validated the effectiveness of VSTJ-based stiffness tuning. Composed of five serially connected VSTJs, TenFiBot can achieve both uniform and non-uniform stiffness distributions. Experimental tests demonstrated that stiffness distribution significantly affects swimming performance: the robotic fish reached a maximum swimming speed of 0.87 BL/s with Strouhal numbers ranging from 0.35 to 0.5. This research provides novel insights into stiffness control for biomimetic robots, and VSTJs can also be extended to applications such as compliant robotic arms and snake-like robots. Subsequently, Chen et al. [93] further advanced this research direction by developing a planar model incorporating the flexibility of soft fish skin and tail, addressing the lack of dynamic models for tensegrity robotic fish. They demonstrated that the rational combination of body stiffness components (tensegrity cables, caudal fin, and fish skin) can significantly enhance swimming performance, and this model provides critical guidance for the optimized design and performance prediction of tensegrity robotic fish.

Antagonistically-controlled variable stiffness structures have emerged as a preferred solution for high-frequency and multi-task biomimetic robotic fish, attributed to their advantages of high biomimetic fidelity and superior efficiency. However, their intricate control logic and inherent energy loss issues restrict their application in low-power consumption and miniaturized robotic systems.

### 3.2. Structurally-Controlled Variable Stiffness Structure

**Figure 5:**
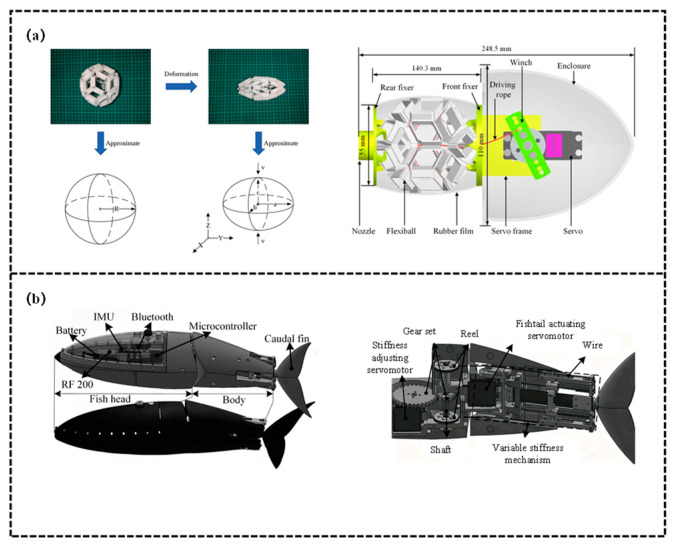
Application of structurally-controlled variable stiffness structure in robotic fish. (**a**) Origami flexiball-inspired soft robotic jellyfish [95]. The red lines in the figure correspond to the driving ropes (three in total), which are wound around the grooves of the origami structure to transmit the tensile force from the servo motor, driving the origami structure to contract and deform, and thus serving as the power transmission medium between the servo motor and the origami structure. The gray rhombic triacontahedron is the flexiball, which forms a sealed variable-volume cavity together with the surface rubber film; during contraction, it squeezes the internal water, generating thrust through jetting via the nozzle, and during expansion, it reverts to its original form by its own elasticity and absorbs water, completing the propulsion cycle. The green zone represents the winch, a mechanical structure that achieves rope winding, releasing, and tension control; the yellowish-green zones are the fixers, mounted at both ends of the origami flexiball respectively and fixed to the origami structure via buckles; the yellow zone is the servo frame. (**b**) Elastic-spine-based variable stiffness robotic fish [96].

Structurally-controlled variable stiffness structures represent another core branch of bio-inspired variable-stiffness structures. Their core principle lies in regulating stiffness by modifying the structure’s geometric shape, geometric arrangement (e.g., angle, topological configuration), or effective force-bearing length—rather than relying on intrinsic material properties—thereby perfectly replicating the structural adaptation mechanisms of natural organisms, as shown in Figure 5. For instance, fish curl their fins into a “cupped shape” during maneuvering or high-load conditions, this increases the second moment of area of the fin cross-section, enhancing flexural stiffness and enabling more efficient hydrodynamic propulsion [94]. Such structures do not depend on complex materials or actuation components, instead, through modular design and geometric reconstruction, they achieve wide-range and rapid stiffness switching, adapting to the mechanical requirements of different swimming scenarios. Hu et al. [95] proposed a soft robot scheme integrating origami metamaterials with jellyfish biomimetics. Leveraging the properties of the rhombic triacontahedral origami metamaterial, radial contraction/expansion of the origami ball is achieved by tightening/relaxing driving ropes via a servo motor. Through the synergy between structural deformation and material elasticity, the robot’s stiffness is dynamically regulated to adapt to jet propulsion and resetting. Targeting the jet propulsion of prolate medusae as the biomimetic objective, the research first selected the rhombic triacontahedron through comparative hydrodynamic simulations, established a jet propulsion model to confirm a nozzle diameter of 15 mm, and adopted an MG996R servo motor combined with three driving ropes for actuation. Experimental validation demonstrated a maximum thrust force of 35.2 mN, an average swimming speed of 11.58 mm/s, a Strouhal number (St) of 0.2159 (within the high-efficiency range), and a cost of transport (COT) of 19.02. Furthermore, the study pointed out the necessity to enhance maneuverability, perceptual capability, and structural integration. Liao et al. [96] proposed an elastic-spine-based variable stiffness robotic fish scheme, aimed at addressing the challenge of fast, online, and large-range stiffness adjustment for robotic fish. Spring steel is adopted to emulate the fish spine, which is divided by a slider into a fully constrained segment (fixed at both ends) and a semi-constrained segment (fixed at one end and free at the other). A stiffness-adjusting servo motor drives the slider to move, altering the effective lengths of the two segments to realize stiffness regulation. The robotic fish can switch stiffness within the range of 17.62–184.7 N/m in 0.26 s, while maintaining a streamlined appearance during the adjustment process. In this study, a Kane-based dynamic model is established to reveal the correlation between propulsive performance and stiffness (the optimal stiffness increases positively with frequency). Subsequently, a stiffness adjustment strategy for multi-stage swimming is constructed to minimize energy consumption and speed error. Finally, simulations and experiments verify that robotic fish can achieve stable and efficient multi-stage swimming.

Structurally-controlled variable stiffness structures have demonstrated favorable applicability under specific flow regimes and operating conditions, attributed to their advantages of concise design and lightweight characteristics. However, their inherent limitations in high-frequency performance and high manufacturing barriers necessitate synergy with other technologies to expand their application scenarios.

### 3.3. Mechanically-Controlled Variable Stiffness Structure

**Figure 6:**
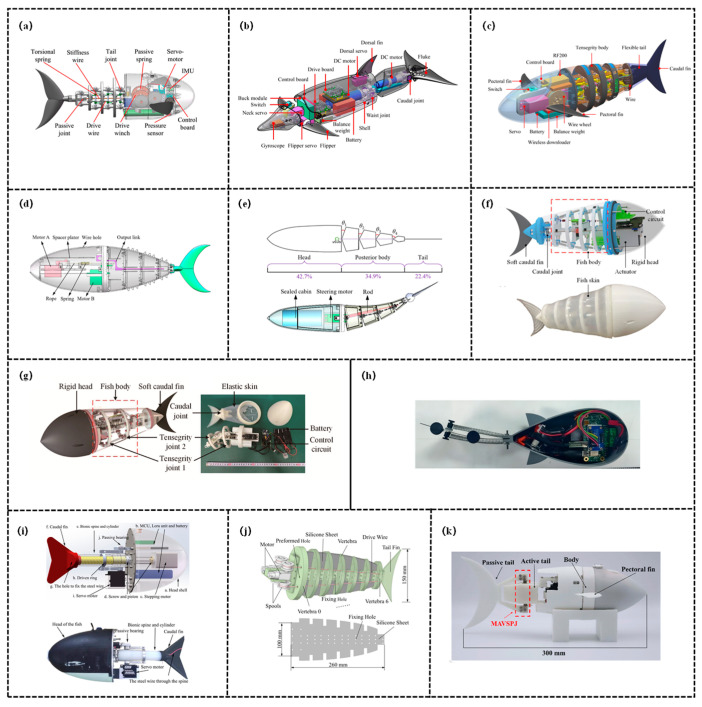
Application of mechanically-controlled variable stiffness structure in robotic fish. (**a**) The tendon-driven robotic (T-Fish) [68]. (**b**) Bionic robotic dolphin [97]. (**c**) Bionic tensegrity robotic fish [98]. (**d**) Online variable stiffness fish-inspired robot (On-VSFR) [99]. (**e**) Robotic tuna with shell-like tensegrity joints [100]. (**f**) Tensegrity fish robot with high-frequency oscillation and online body stiffness adjustability (TenFiBot-HFS) [101]. (**g**) The tensegrity bistable robotic fish (TenFiBot-BIS) [102]. (**h**) Fish-like robot with a bistable tail [103]. The main body adopts a streamlined extruded hydrofoil design, with core electronic components such as a brushless motor, a controller, an IMU, and a battery compactly arranged in the head, while a 0.64 mm-thick bistable tail fin made of PETG plastic is assembled at the rear end. (**i**) Variable stiffness robotic fish with a compressible flexible bionic spine [104]. (**j**) Wire-driven bionic fishtail actuator [105]. (**k**) Robotic fish with modular adaptive variable stiffness passive joint (MAVSPJ) [106].

Mechanically-controlled variable stiffness structures do not rely on changes in the physical properties of smart materials, instead, dynamic stiffness adaptation is realized through the mechanical synergy of purely mechanical structures, as shown in Figure 6. Such structures replicate the synergistic regulation mechanism of the tendon-muscle-skeleton system in biological organisms, offering advantages of high structural reliability, low energy consumption, and stable response. They are widely adaptable to the propulsion and maneuvering requirements of biomimetic robotic fish across different scales.

There are two primary approaches for stiffness regulation in mechanically-controlled variable stiffness structures: one involves adjusting the equivalent stiffness of the system by modifying the pre-tension or preload state of elastic components such as springs and elastic strings. Qiu et al. [68] designed a tendon-driven robotic fish T-Fish and proposed locomotion optimization methods to enhance its maneuverability and stability. Its stiffness-tuning mechanism is as follows: a variable-stiffness passive caudal fin is constructed using steel wire ropes and springs, and a servo motor is employed to adjust the pretension of the passive spring—these further changes the passive joint torque, thereby indirectly adapting to the stiffness requirements of different swimming states. Experimental validation demonstrates that this scheme achieves remarkable effects and provides valuable references for the structural design and motion control of bio-inspired underwater robots. Chen et al. [97] proposed an active variable stiffness control scheme for a bionic robotic dolphin. Specifically, its stiffness-tuning mechanism operates as follows: the cycle-synchronous torque (CST) mode of the caudal joint motor is utilized to mimic the characteristics of a torsional spring, after setting the stiffness coefficient, the target torque is calculated in real time based on the joint angle measured by an encoder, and the motor output torque is controlled in a closed loop (τ = Ksθ), thereby actively adjusting the stiffness of the caudal joint. The study explored the influences of frequency and stiffness on swimming performance, and proposed an online optimization scheme—searching for the optimal stiffness under different frequencies via the model and adjusting it in real time. Experimental results demonstrated that this scheme achieves remarkable effects and provided a new approach for optimizing the performance of underwater robots through stiffness adjustment. Subsequently, Chen et al. [98] proposed a novel design scheme for a bionic robotic fish based on a tensegrity structure. Specifically, its stiffness-tuning mechanism is as follows: two servo motors synchronously drive the wires on both sides of the fish body to maintain a consistent wire contraction length, this further modifies the pretension of longitudinal and transverse springs in the tensegrity structure, thereby realizing online adjustment of the fish body stiffness—with a larger contraction length leading to higher body stiffness. The study explored the effects of oscillation frequency, amplitude, and stiffness variation on swimming performance. Experimental results demonstrated that this scheme achieves remarkable effectiveness. This provides a new direction for the design of bionic robotic fish that simultaneously integrates continuum characteristics and variable stiffness functionality. Liu et al. [99] published a study, developing a novel fish-inspired robot with variable stiffness capability to investigate the influence of stiffness on swimming performance. Two types of variable stiffness mechanisms are proposed: the offline variable stiffness (Off-VSFR) adjusts stiffness by manually modifying the pre-stretching length or type of the spring, the online variable stiffness (On-VSFR) is based on a MACCEPA variant, where a steering motor is integrated to control the spring preload, achieving wide-range online stiffness regulation. Both mechanisms feature the advantages of decoupling between stiffness and rotation angle, simple structure, and low energy consumption. Inspired by real fish that enhance swimming speed and efficiency through stiffness adjustment, the tuna was selected as the biomimetic object. A shell-like structure was adopted to replace the wrinkle-prone elastic skin, and tensegrity joints were employed to reduce frictional loss. Pool experiments were performed, and the collected data were processed using the undulatory reconfiguration technique. The results indicate that the rotational stiffness of the robot body must match the driving frequency (increasing as the frequency rises), and the body stiffness serves as a key factor in maintaining stable stride length. Liu et al. [100] addressed the issues of high frictional loss and insufficient internal space in traditional robotic fish by developing a robotic tuna with shell-like tensegrity joints. Through single-motor multi-joint actuation and parameter optimization, the swimming performance of the robot was significantly enhanced. This study presents a design of the robotic tuna based on shell-like tensegrity joints, which consist of compressed rods and tensioned cables. The joint stiffness is modified by adjusting the pretension of tension elements (e.g., elastic strings). Compared to tendon-driven and wire-driven counterpart robotic fish, the proposed robotic tuna exhibits superior performance. Ding et al. [101] proposed a high-frequency oscillating tensegrity robotic fish (TenFiBot-HFS) with online adjustable full-body stiffness, addressing the issues of low frequency and mostly offline caudal fin stiffness adjustment in existing variable-stiffness robotic fish. Its variable stiffness mechanism is based on tensegrity joints and servo control: the trunk incorporates two variable-stiffness tensegrity joints, each adjusting the pre-stretch of spring S via variable stiffness actuators (VSAs). By combining the elastic force of the spring and the joint resistance torque, a wide range of rotational stiffness adjustment for the joints is achieved and an adjustment response time of approximately 0.12 s. The full-body stiffness distribution can be dynamically adjusted online by altering the stiffness combination of the two joints. The study verified the effectiveness of the joint variable stiffness, and its variable stiffness capability is superior to that of most similar robotic fish, providing insights for the dynamic performance optimization of high-frequency robotic fish. Chen et al. [102] proposed a rigid-soft coupled biomimetic robotic fish (TenFiBot-BIS) based on the tensegrity principle, addressing the issue that existing bistable robotic fish are classified as purely soft or rigid and thus performance-constrained. This design enables adjustable bistable characteristics and improved swimming performance. TenFiBot-BIS adjusts the preload of spring S via Motor 2 to modify the system’s energy barrier (ΔE), thereby adapting to the dynamic stiffness required for bistable oscillation. The tensegrity structure achieves rigid-soft coupling, and when combined with intermittent gear transmission, the robotic fish can match hydrodynamic requirements through energy parameter regulation under different operating conditions. The study designed a rigid-soft coupled structure: the rigid head houses the circuit, the body is a tensegrity system, and the soft caudal fin is made of liquid silicone rubber. Equipped with two servo motors (Motor 1 for gear driving and Motor 2 for adjusting bistable parameters), the robot adopts intermittent gear transmission to accommodate bistable snap-through.

The other approach involves regulating the system stiffness by modifying the geometric layout, connection mode, or overall structural topological configuration of elastic components. Chivkula et al. [103] designed a single-actuator underactuated fish-like robot, which achieves gait switching via a bistable tail. This design addressed the issue of poor turning performance in traditional rotor-driven fish-like robots, providing a solution for agility optimization of underactuated underwater robots. The study realized passive stiffness adjustment of the tail through a bistable structure: the tail comprises a PETG plastic plate and pre-stretched springs mounted on both sides, forming an elastic potential energy structure with double wells. The straight tail configuration is unstable, while the leftward and rightward deflected configurations are stable. By regulating the frequency and amplitude of the internal rotor, the tail can be controlled to switch between single-well oscillations and inter-well oscillations. This design enables the effective stiffness of the tail to dynamically change according to actuation conditions: high frequency with low amplitude triggers single-well oscillations to achieve turning motion, while low frequency with high amplitude induces inter-well oscillations to realize straight-line motion. Zhu et al. [104] proposed a variable stiffness fishlike propeller with a compressible flexible bionic spine. The bionic spine is fabricated from silicone, and its stiffness is dynamically adjusted by compressing or releasing the spine—driven by a piston connected to a lead screw actuated by a stepping motor. Inspired by pomfrets, the study designed a 40 cm-long robotic fish, derived a method for calculating the stiffness of periodically heterogeneous beams, and established a dynamic model using the pseudo-rigid body model and the Lagrangian method. Simulations revealed that fixed stiffness corresponds to a specific optimal frequency band, while variable stiffness must increase with frequency to sustain high efficiency. Experimental results verified the stiffness model with an error of only 3.73%: low stiffness is suitable for low frequencies, high stiffness for high frequencies, and variable stiffness can maintain a low Cost of Transport (COT) over a wide frequency range. This work provides a novel solution for the efficient locomotion of robotic fish. Luo et al. [105] proposed a wire-driven flexible bionic fishtail actuator to investigate its propulsive performance and C-Start escape maneuver capabilities. By optimizing the combination of drive wires, three schemes were designed: inelastic wire-driven (W), elastic wire-driven (We), and “inelastic wire + additional elastic wire” (Wie). The elastic wire stores energy through stretching and releases energy to provide assistance, thereby regulating the stiffness characteristics of the tail’s motion. Inspired by the escape behavior of fish, a bionic fishtail consisting of a drive section, an actuator section, and a caudal fin was designed, periodic oscillation of the fishtail was achieved by controlling the contraction of the drive wires via a proportional-integration-differentiation (PID) controller. Experiments were conducted under two modes: bilateral large flapping angle mode and unilateral infinite bending mode (C-Start), aiming to compare the thrust performance of different stiffness and drive wire combinations. The results indicate that the thrust of the inelastic wire is significantly superior to that of the elastic wire, while the additional elastic wire can slightly enhance the thrust. Moreover, the spine stiffness affects the bending angle and thrust stability: lower stiffness is more prone to forming a C-shaped configuration, whereas higher stiffness exhibits stronger resistance to fluid drag. This study provides a novel approach for the design of high-efficiency propulsion and escape maneuvers in bionic fish. Wang et al. [106] proposed a robotic fish scheme incorporating a modular adaptive variable stiffness passive joint (MAVSPJ), addressing the issue that existing variable-stiffness tail structures of robotic fish require additional driving sources and thus increase the complexity of design and control. Its variable stiffness mechanism is based on torsion springs and linear springs: At low frequencies, the phase difference of the passive tail is small, the trigger does not contact the moving plate, the spring force, and the joint stiffness remains unchanged. At high frequencies, the phase difference of the passive tail increases, the trigger pushes the moving plate to elongate the spring, generating a spring force to enhance the joint stiffness. Furthermore, the stiffness variation process can be customized by adjusting spring parameters and the trigger angle.

Mechanically-controlled variable stiffness structures have met the stiffness regulation requirements under medium-low speed and stable operating conditions, attributed to their advantages of low energy consumption and high structural reliability. However, their inherent limitations in adjustment range and fatigue resistance render them more suitable for long-duration operation scenarios where the amplitude of stiffness switching is not strictly demanded.

## 4. Fluid-Driven Variable Stiffness Structure

### 4.1. Pneumatic Variable Stiffness Structure

**Figure 7:**
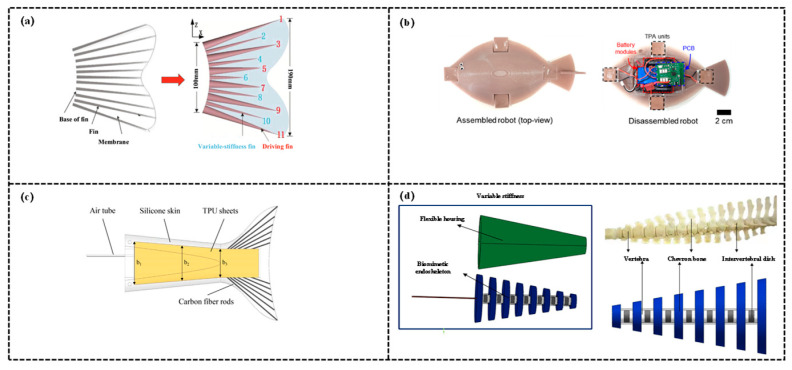
Application of pneumatic variable stiffness structure in robotic fish. (**a**) robotic fish with a variable stiffness pneumatic soft bionic caudal fin [107]. Red markers denote the driving fins (6 in total), numbered 1, 3, 5, 7, 9 and 11 in the figure, which serve as the power actuation components enabling the bending and oscillation of the caudal fin, while blue markers represent the variable-stiffness fins (5 in total), numbered 2, 4, 6, 8 and 10 in the figure, acting as the core functional components that realize the dynamic stiffness regulation of the caudal fin. (**b**) Biomimetic flat fish robot (Flatfishbot) [108]. (**c**) Robotic fish with a variable-stiffness tail based on layer-jamming technology [109]. The yellow part represents the TPU sheets, serving as the core functional layer for variable stiffness that achieves stiffness regulation through layer-jamming; the sheets are trapezoidal in shape, with their number gradually decreasing along the tail length (8 layers → 6 layers → 4 layers), forming the optimal stiffness distribution of “stiff at the root and soft at the tip”. The black lines denote the carbon fiber rods, which act as rigid supporting components at the upper and lower edges of the caudal fin, addressing the issue of excessive edge softness caused by insufficient filling of TPU sheets, oscillating synchronously with the middle TPU sheets to enhance propulsion efficiency. (**d**) Pneumatic Variable Stiffness Dolphin-like Tail Actuator (PVSA) [110]. The white part consists of serially connected vertebrae that form the rigid supporting skeleton of the tail, with stiffness distributed in a gradient along the length; the gray part represents intervertebral discs, which act as flexible connective tissues to absorb impacts and adapt to bending; the blue part denotes chevron bones that enhance muscle attachment and restrict excessive bending.

In pneumatic variable-stiffness robotic fish, the core mechanism for stiffness adjustment involves dynamically modifying the degree of chamber expansion and structural supporting force through the inflation/deflation of specific elastic chambers (e.g., dual chambers of driving fins, particle-filled chambers of variable-stiffness fins, thermoelectric-pneumatic phase-change chambers), thereby ultimately achieving precise regulation of stiffness, as shown in Figure 7.

Notably, under most operating conditions, the inflation volume exhibits a positive correlation with stiffness—greater inflation leads to more significant chamber expansion, stronger supporting force exerted on surrounding structures, and consequently higher overall stiffness. Cheng et al. [107] focused on the design, modeling, and finite-time tracking control of a variable stiffness pneumatic soft bionic caudal fin. Stiffness adjustment is achieved via jamming technology: particles are filled into the internal chambers of the caudal fin to modulate stiffness. Through experimental tests around 2020, under negative pressures of −40 kPa and −80 kPa, the variable stiffness columns filled with 2 mm annular particles exhibited the optimal stiffness variation effect. The negative pressure of the chambers can be regulated by a vacuum pump to alter the particle state, enabling dynamic adjustment of the caudal fin’s stiffness. The study designed a bionic caudal fin comprising 6 driving fins and 5 variable stiffness fins, and conducted bionic experiments. Experimental results confirmed that the thrust increases as the air pressure of the driving fins rises, while the thrust decreases as the negative pressure of the variable stiffness fins increases. Simulations demonstrated that the system output can quickly and accurately track the desired trajectory, validating the effectiveness of the proposed design and control method. Lee et al. [108] designed an untethered pneumatic biomimetic soft robotic fish (Flatfishbot) that mimics the gliding locomotion of marine vertebrates. Flatfishbot achieves stiffness variation by leveraging the liquid-vapor phase transition of thermoelectric pneumatic actuators (TPAs) and the properties of elastic materials: an external control system actively reverses the current direction to trigger the Peltier effect—the heating mode vaporizes the fluid, leading to chamber expansion and increased local buoyancy, the cooling mode reverses the current to condense the vapor into liquid, resulting in chamber contraction and reduced buoyancy. The core objective of the research is to develop a high-efficiency gliding robotic fish. By controlling the alternating heating/cooling of TPAs, it emulates the intermittent locomotion of marine organisms (“active ascent—gliding descent”), achieving an average speed of 0.515 BL/s. The robotic fish is capable of turning, transporting 70 g cargo, and carrying a waterproof camera for underwater monitoring. Hong et al. [109] proposed a variable-stiffness tail for robotic fish based on layer-jamming technology, addressing the issues of significant shape deformation and narrow stiffness range in traditional designs. This study dynamically adjusts stiffness via a layer-jamming structure: the variable-stiffness tail is encapsulated in silicone skin and integrates trapezoidal thermoplastic polyurethane (TPU) sheets. The number of TPU layers gradually decreases from 8 at the front end to 4 at the distal end, forming a conical distribution to maintain optimal stiffness. Built-in carbon fiber rods enhance structural stability. By regulating the internal cavity vacuum pressure (0~30 kPa) with a vacuum pump, the TPU sheets are compressed under atmospheric pressure, switching among three phases—pre-slip (maximum stiffness), partial-slip (decreasing stiffness), and full-slip (minimum stiffness)—achieving an approximately 10-fold stiffness variation. Notably, the tail shape remains unchanged during adjustment, ensuring hydrodynamic performance. Experimental validations were conducted: (1) Constant load tests revealed that stiffness increases significantly as vacuum pressure decreases, (2) Underwater oscillation tests demonstrated that the model error is mostly less than 20%, (3) Thrust tests indicated that higher frequencies require corresponding higher vacuum pressure (stiffer tails). At 1.5 Hz, the net thrust reaches 292.3 mN under 20 kPa. These results confirm that the tail can assist robotic fish in maintaining efficient swimming over a wide frequency range, providing a novel solution for the development of underwater biomimetic robots. Zhang et al. [110] addressed the issues of low propulsion efficiency and high noise in traditional underwater robots. By integrating soft robotics technology with the biological characteristics of dolphins, they developed a Pneumatic Variable Stiffness Dolphin-like Tail Actuator (PVSA) and conducted its performance analysis. The PVSA is composed of a pneumatic bidirectional bending soft actuator and a cable-driven variable stiffness mechanism connected in series: The actuator achieves dolphin-like dorsoventral movement by regulating the air pressure difference between the upper and lower wrinkled chambers, and the lacunar ratio (s = 0.5) was optimized through numerical simulation to ensure deformation performance, Based on the particle interference principle and the spinal characteristics of dolphins, the variable stiffness mechanism adopts a hybrid particle chain structure consisting of series-connected rigid particles and silicone rubber sheets. In experiments, adjusting the cable tension caused particle compression and an increase in the radial dimension of the compressed flexible sheet, thereby enhancing the bending stiffness. Shore A 15° silicone sheets were selected to fabricate the flexible sheets, whose stiffness adjustment range exceeds 30 times their own base bending stiffness with minimal change in total length. The study analyzed the bending behavior of the PVSA using a pseudo-rigid-body model and built an underwater experimental platform. The results indicate that different motion frequencies correspond to different stiffness values for maximum thrust, with higher stiffness required for high-frequency motion, the PVSA can enhance thrust by adjusting its stiffness, maintains its shape during stiffness variation to avoid thrust loss, and provides a reference for underwater biomimetic propulsion technology.

Pneumatic variable stiffness structures have emerged as a crucial option for multi-scenario adaptation of medium-to-large-sized biomimetic robotic fish, attributed to their advantages of a wide adjustment range and rapid response. However, their reliance on sealing integrity and inherent limitation in untethered endurance render them more suitable for scenarios without deep-sea or long-endurance requirements, such as nearshore monitoring and short-duration operations.

### 4.2. Hydraulic Variable Stiffness Structure

**Figure 8:**
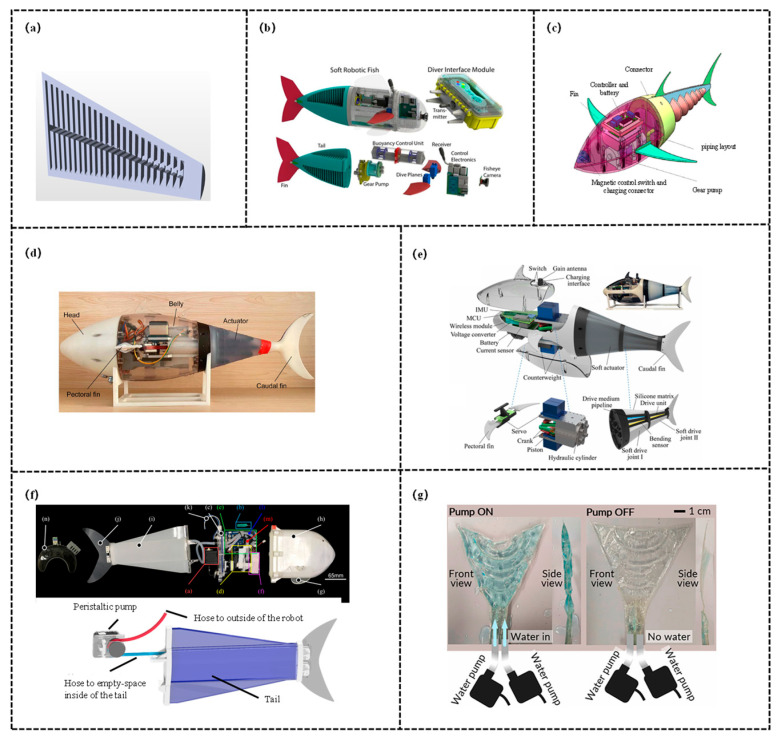
Application of hydraulic variable stiffness structure in robotic fish. (**a**) Novel robot fish with compliant fluidic actuator as a tail (FTFA) [111]. (**b**) Fish Robot with Soft Fluidic Actuation (integrated with Fish Tail Fluidic Actuator, FTFA) [112]. (**c**) Flexible collapsible fluid-driven bionic robotic fish [113]. (**d**) A hydraulic autonomous soft robotic tuna (HasorTuna) [114]. (**e**) Locomotion performance optimization of HyperTuna [115]. (**f**) HVS soft robotic fish [116]. Red frame a: Caudal fin oscillating actuator (XW540-T140-R); Dark blue frame b: Bluetooth receiver (BT-410); c: Plastic body sealing part; Yellow frame d: Actuator (MX-28AT) and peristaltic pump head for the HVS mechanism; Green frame e: Controller (OpenCR1.0); Purple frame f: 11.1 V 1000 mAh 10C lithium polymer battery; g: Pectoral fin; h: Plastic head sealing part; i: Caudal fin integrated with the HVS mechanism; j: Passive caudal fin with fixed stiffness; k: External power cable; l: Connecting pipe to the inner cavity of the caudal fin; Red line m: External connecting pipe of the robot; n: Bluetooth transmitter (RC-100B, BT-410) (**g**) Online hydraulic stiffness-modulated tail [117].

In hydraulic variable-stiffness robotic fish, the core mechanism for stiffness adjustment involves dynamically modifying the degree of chamber expansion and structural supporting force through the inflation/deflation of customized elastic chambers (e.g., embedded fluid channels, origami-structured chambers, McKibben muscle chambers) with hydraulic oil (or functional fluids), thereby ultimately achieving precise and dynamic regulation of stiffness, as shown in Figure 8. Notably, this process requires adaptation to both the chamber structural design and swimming scenarios, furthermore, the fluid volume, pressure, and stiffness generally exhibit a positive correlation, while some designs can realize nonlinear adjustment of stiffness through the optimization of chamber morphology.

Elastic chambers of the embedded fluid channel type exist as fluid channels integrated into the robotic fish body. By regulating the hydraulic oil pressure/flow rate within the channels, the degree of channel expansion and structural supporting force are modified, thereby achieving stiffness adjustment. Haji [111] proposed a novel robotic fish with a compliant fluidic actuator (Fish Tail Fluidic Actuator, FTFA) as its caudal fin, addressing the issues of complex structure, high energy consumption, and low efficiency associated with traditional multi-rigid-link robotic fish. Variable stiffness is achieved via the FTFA: it allows adjusting fluid viscosity, input pressure, channel position and orientation, or regulating the tail’s density and elasticity (e.g., Young’s modulus), thereby altering the overall stiffness of the system. This ultimately adjusts the resonant frequency, enabling efficient swimming control of the robotic fish. Bamdad et al. [112] proposed a design and analysis scheme for a fish robot with soft fluidic actuation, addressing the limitation of constrained maneuverability in a single plane of traditional robotic fish. Dynamic stiffness adjustment is achieved via soft fluidic actuators (SFAs): parallel fluid channels are embedded in the neutral plate of the fish tail, and bending moments are generated by regulating the fluid pressure within the channels to tune the tail stiffness. The designed robotic fish integrates a rigid body and a Fish Tail Fluidic Actuator (FTFA), with upper and lower nozzles mounted at the tail end to enable 3D motion without relying on additional propulsion systems. A tail deflection model is established based on the Euler–Bernoulli beam theory, and an Approximate Analytical Method (AAM) is proposed to balance modeling accuracy and computational efficiency. Simulations and experimental validations demonstrate: Channel cross-section optimization identifies the square cross-section as optimal, which enhances tail deformation by 65% compared to the semicircular cross-section, The robotic fish can achieve 3D trajectory motion, with matched average speed and thrust under steady-state conditions. This work provides an efficient and low-power design and modeling approach for miniature underwater environmental monitoring robots.

Origami-structured chambers alter the degree of chamber expansion via the inflation/deflation of hydraulic oil, thereby regulating the structural supporting force and stiffness while achieving a balance between lightweight design and large deformation capability. Xia et al. [113] focused on the performance optimization of hydraulic-driven biomimetic robotic fish. The study primarily addresses the drawbacks of traditional hydraulic-driven biomimetic caudal fins, including high driving pressure, significant radial expansion, and low energy efficiency. To overcome these defects, the paper proposes two core variable stiffness methods: Firstly, a highly flexible origami-structured caudal fin is adopted. Origami technology is utilized to constrain radial expansion, enabling the injected liquid to be prioritized for conversion into axial bending, achieving a 92.3% energy saving compared to traditional caudal fins. Secondly, a sandwich-structured bionic hybrid neutral layer of the caudal fin is designed via a knitting method. A 0.5 mm epoxy fiberboard is cut into 10 segments, connected by knotting nylon ropes, and the central slit is filled with 1 mm liquid silicone rubber, forming a “sandwich + knot” structure. This structure retains non-stretchability while enhancing flexibility, realizing a 56.7% energy saving compared to the rigid neutral layer.

McKibben artificial muscles serve as elastic chambers, hydraulic oil pressurization induces radial expansion and axial contraction of the muscles, generating structural supporting force and driving force to achieve dynamic stiffness adaptation. Liu et al. [114] proposed a Hydraulic Autonomous Soft Robotic Tuna (HasorTuna), addressing the issues of low frequency and poor endurance of traditional hydraulic robotic fish. HasorTuna achieves dynamic stiffness adaptation through the synergy between drive control and structure. Mimicking the red muscle activation characteristics of tuna, HasorTuna incorporates a gradient duty cycle of 0.22–0.5 T: low duty cycles are adapted to low frequencies, while high duty cycles suit high frequencies, with equivalent stiffness adjusted by modifying the pressurization duration of the driving units within a single cycle. Additionally, via hydraulic-structural coupling, a double-cylinder plunger pump independently controls the pressure of the McKibben artificial muscle driving units on both sides, paired with an oblate-structured soft actuator (silicone matrix + artificial muscles). Pressure changes regulate the bending stiffness of the actuator, indirectly optimizing the tail propulsive stiffness. Experimental results demonstrate: excellent speed at low frequencies and low duty cycles (0.5 BL/s), the highest speed of 0.84 BL/s and the minimum cost of transport (COT) of 11 J/(kg·m) at high frequency and high duty cycle. HasorTuna is capable of 3D motion, providing a solution for high-efficiency underwater biomimetic robots. Subsequently, Liu et al. [115] proposed a hydraulically powered double-joint soft robotic fish (HyperTuna) and a locomotion optimization scheme, addressing the challenges in the design and control of multi-joint hydraulically powered soft robotic fish. HyperTuna employs McKibben artificial muscles as driving units, which are arranged in pairs on both sides of the body axis. A four-cylinder piston pump provides pressure pulses of hydraulic oil (L-HM46), servo motors control piston movement via crank-slider mechanisms, regulating oil pressure to induce radial expansion and axial contraction of the muscles. This in turn alters the joint bending angle to achieve stiffness modulation. Flexible bending sensors are embedded in the neutral layer of the joints, combined with a closed-loop control system integrating a Central Pattern Generator (CPG) and a Proportional-Integral-Derivative (PID) controller, the robot accurately perceives and adjusts stiffness, ensuring motion stability.

Other elastic chambers feature customized flexible structures (not belonging to the aforementioned typical types). By adjusting the inflation/deflation of hydraulic oil or water to alter the chamber volume and expansion degree, the structural supporting force and stiffness are regulated to adapt to specific swimming scenarios. Ju [116] proposed a Hydraulic Variable Stiffness (HVS) mechanism, addressing the issues of complex multi-joint structure and fixed caudal fin stiffness in traditional robotic fish and achieving the optimization of swimming locomotion. The robotic fish relies on the HVS mechanism to realize stiffness variation—regulating the water volume in the caudal fin via a peristaltic pump to alter the fin thickness: when the water volume increases, the caudal fin thickens and stiffness rises, when the water volume decreases, the fin thins and stiffness reduces. The study designed an autonomous soft robotic fish, integrating components such as a peristaltic pump and a battery, which can operate independently for 40 min. A Pseudo-Rigid-Body (PRB) 3R model was established for modal analysis, revealing that the optimal caudal fin stiffness increases with the elevation of driving frequency. Obayashi et al. [117] addressed the challenge of optimizing the thrust and efficiency of soft robotic fish, proposing a novel online hydraulic stiffness modulation scheme. Stiffness modulation is achieved via a fluid-driven mechanism: a patterned pouch fabricated from a vacuum-sealed bag is encapsulated in a silicone layer. Dynamic stiffness control of the caudal fin is realized by inflating and deflating the pouch using two water pumps with a flow rate of 240 L/h (inflation completed in 0.15 s and instantaneous deflation upon pressure release). This enables soft-to-stiff switching within a single tail-beat cycle, with a stiffness response speed of 0.15 s—outperforming traditional schemes such as shape memory alloys (SMA, 10–40 s) and structurally controlled spines (0.26 s).

Hydraulic variable stiffness structures have satisfied the stiffness regulation requirements of high-performance biomimetic robotic fish, attributed to their characteristics of high precision, rapid response, and strong load-bearing capacity. However, their complex system design and high maintenance costs render them more suitable for professional scenarios demanding high motion precision and load capacity, and impede their popularization in miniaturized and low-cost robotic systems.

## 5. Hybrid-Driven Variable Stiffness Structure

**Figure 9:**
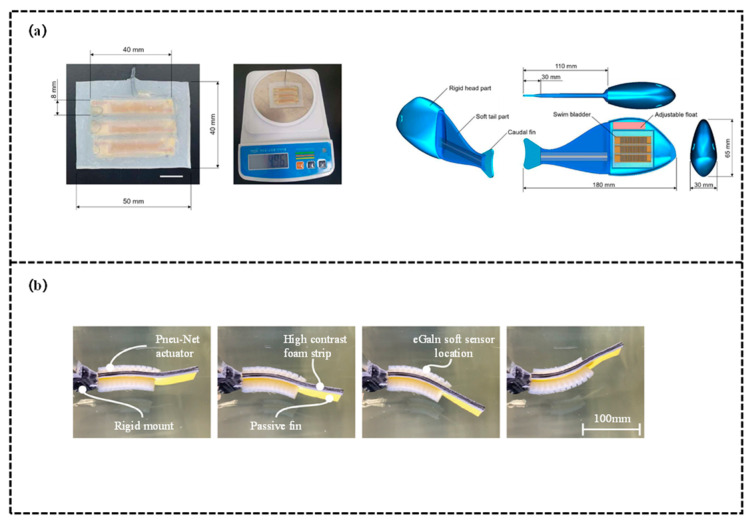
Application of hybrid-driven variable stiffness structure in robotic fish. (**a**) Intelligent material and fluid-driven variable stiffness structure. Robotic soft swim bladder using liquid-vapor phase transition [118]. The left panel specifies the exact geometric dimensions and weight parameters of the fabricated soft swim bladder, while the right panel illustrates the overall dimensions, composition of core components and spatial layout of the biomimetic fish robot, clearly presenting its structural design consisting of a rigid head and a soft tail. (**b**) Bio-inspired and fluid-driven variable stiffness structure. Soft robotic fish with integrated soft sensing [119]. This panel presents a physical schematic of the pneumatically actuated soft robotic fish swimming in a water tank, with the core constituent components and the layout of the soft sensors clearly labeled.

Composite-driven variable stiffness structures integrate two or more driving modes/stiffness regulation mechanisms, achieving synergistic optimization of stiffness adjustment range, response speed, and control precision through multi-field coupling or structural collaboration, as shown in Figure 9.

This category fully leverages the complementary advantages of different single mechanisms, effectively addressing the performance bottlenecks of individual schemes and expanding the adaptive capacity of robotic fish in complex underwater scenarios. Kang et al. [118] designed a robotic soft swim bladder focusing on liquid-vapor phase transition. The core variable stiffness mechanism is as follows: elastic buoyancy pouches are fabricated from TPU films, each filled with 0.08 mL of liquid-vapor phase transition material. Resistive heating elements heat the material to convert it from liquid to vapor, causing the buoyancy pouches to expand and the supporting force to increase, thereby achieving stiffness enhancement, upon cooling, the material reverts to a liquid state, the buoyancy pouches contract, and the stiffness decreases, completing the stiffness modulation. In terms of performance, an optimal heating current of 0.8 A and an intermittent current strategy were adopted to extend the service life of the buoyancy pouches. Test results indicate that the soft swim bladder adjusts buoyancy by activating 1–3 buoyancy pouches, corresponding to different motion modes of the robotic fish. When the tail is driven with specific parameters, the forward swimming speed reaches approximately 28.8 mm/s, and the robotic fish can also perform composite motions, providing a solution for underwater robot control. Lin et al. [119] focused on the undulatory locomotion control of a subcarangiform soft robotic fish, proposing a data-driven modeling and closed-loop control scheme to achieve synergistic control of fully soft actuators and sensing. Variable stiffness is realized through the co-contraction of antagonistic actuators by adjusting the air pressure overlap ratio on both sides (ranging from −10% dead time to 10% overlap). A higher overlap ratio results in greater stiffness, which can reduce the maximum swing rate of lateral movement and dynamically adapt to the requirements of different motion scenarios. The study employs antagonistic fast-PneuNet pneumatic actuators (silicone material) to drive the robotic fish, with eutectic gallium-indium (eGaIn) embedded in silicone channels as strain sensors. A lumped parameter model was developed, simplifying the robotic fish into a rigid link-hinge system, and parameter tuning was performed using the obtained experimental data. This model can accurately predict the behavior of the robotic fish over ranges of different driving frequencies and pressure amplitudes. Based on the model, a PID amplitude controller was designed, which realizes closed-loop control by extracting the tail-beat amplitude in real time through soft sensing.

Hybrid-driven variable stiffness structures have emerged as a pivotal direction for performance breakthroughs of biomimetic robotic fish in complex scenarios, attributed to their advantage of multi-mechanism synergy. However, their intricate system design and relatively high engineering costs render them more suitable for professional fields with stringent performance requirements at present, and it is necessary to reduce the application threshold through lightweight design and control algorithm optimization.

## 6. Discussion

Table 1 summarizes the swimming performance of different types of robotic fish. Max. Speed refers to the highest forward velocity achievable by biomimetic robotic fish during stable locomotion under a specific driving frequency. Two units are commonly used in the study: “m/s” and “BL/s”. The latter eliminates the interference of prototype size differences on speed comparison, enabling more objective performance evaluation across different-scale robotic fish. Min. Radius denotes the smallest circular trajectory radius that a robotic fish can achieve when executing a turning maneuver. It is typically expressed in the dimensionless unit “BL (body length)” and serves as a key indicator of maneuverability. A smaller value indicates greater turning flexibility, making the robotic fish well-suited for operations in confined spaces. Max. Turn Rate represents the angular change completed by a robotic fish per unit time during turning, with the unit “°/s”. As a critical metric for assessing turning responsiveness and agility, it directly reflects the robotic fish’s ability to adjust direction rapidly. In the aquatic animal’s locomotion, the Strouhal Number, *SN*, is defined as:

$$SN=\frac{fA_{p-p}}{U}$$

where *f* is the frequency, *A_p-p_* is the peak-to-peak amplitude at the tail end, *U* is the cruising speed. Most of the aquatic animals have their Strouhal Numbers falling in the narrow range between 0.2 and 0.4, and the efficiency is high within this range.

The maximum instantaneous speed in the table is achieved by a small-sized robotic fish driven by smart materials: the soft robotic fish [70] can reach 0.05 m/s at a driving frequency of 100 Hz, significantly outperforming other types. Among medium-to-large robotic fish, the bio-inspired mechanically controlled fish-like robot with a bistable tail [103] exhibits outstanding performance, attaining a speed of 0.5 m/s (2.1 BL/s) at 3 Hz, while the hydraulically driven HyperTuna [115] reaches 0.58 m/s (1.12 BL/s) at 6 Hz. The optimal speed of most robotic fish ranges from 0.01 to 0.74 m/s (0.1 to 1.423 BL/s), with tuna-inspired robotic fish generally demonstrating superior performance. For instance, the hydraulically driven HasorTuna [114] achieves 0.48 m/s (0.84 BL/s) at 4 Hz and is capable of stereoscopic maneuvering. Notably, other robotic fish also possess 3D locomotion capabilities: the pneumatically driven Flatfishbot [108] can perform complex actions such as turning and cargo transportation. Regarding driving frequency adaptability, it maintains an average speed of 0.515 BL/s even at a low frequency of 1.5 Hz. In contrast, the mechanically controlled TenFiBot-HFS [101] reaches 0.41 m/s (1.13 BL/s) at a high frequency of 5 Hz. Soft robotic fish (e.g., SoRoFAAM-1 [85]) feature a minimum turning radius of 0.15 BL, despite their relatively low speed, their flexibility renders them well-suited for operations in confined spaces. The Strouhal numbers of most robotic fish fall within the range of 0.2159–1.1. Among these, the TenFiBot series [71] (0.35–0.5) and HasorTuna [114] (0.6–1.1) operate within the high-efficiency swimming interval, validating the optimization effect of stiffness adjustment and frequency matching. Hybrid-driven robotic fish achieve a balance of multiple performance metrics. For instance, the soft robotic fish integrated with soft sensing can realize closed-loop control of the tail-beat amplitude through real-time perception, thereby enhancing motion stability. As illustrated in Table 2, the average value of the maximum swimming speed for each type of robotic fish are presented. It is seen that the bio-inspired robotic fish attains the highest swimming velocity, reaching 1.116 BL/s. This is followed by the fluid-driven type (0.641 BL/s) and the smart material type (0.364 BL/s). In contrast, the hybrid-driven robotic fish exhibits the lowest swimming performance, with a measured speed of merely 0.16 BL/s.

Table 3 summarizes the advantages, disadvantages, and application scenarios of various types of variable-stiffness structures. Among smart material-driven types, thermally responsive ones (SMP, SMA) offer advantages of stable material properties and suitability for miniaturization but suffer from difficulties in temperature control and slow response, making them suitable for miniature underwater monitoring and low-speed stable propulsion; electrically responsive ones (PZT) feature fast response and high propulsion efficiency yet are limited by small output displacement and high driving voltage, which are applicable to narrow-space operations and high-frequency micro-maneuvering. Within bio-inspired types, antagonistically-controlled structures exhibit high biomimetic fidelity and superior efficiency but entail high energy consumption and complex control, being well-suited for high-frequency swimming robots and marine environment detection; structurally-controlled ones boast strong material compatibility and lightweight design, while their advantages in intermediate flow regimes (1 ≤ Re ≤ 2000) remain unclear and they require high processing precision, thus suitable for specific flow regime operations and low-energy consumption tasks; mechanically-controlled ones are characterized by simple structure and low energy consumption but have a narrow stiffness adjustment range and are prone to fatigue after long-term use, which are applicable to medium-low speed stable swimming and long-endurance underwater monitoring. For fluid-driven types, pneumatic structures provide a wide stiffness adjustment range and excellent hydrodynamic performance but face challenges in underwater sealing and short untethered endurance, making them suitable for nearshore monitoring and low-speed gliding; hydraulic structures deliver fast response, precise regulation, and strong load-bearing capacity yet suffer from complex systems and high maintenance costs, which are applicable to deep-sea professional operations and high-precision maneuvering. Hybrid-driven structures, while integrating the advantages of multiple mechanisms, adapting well to complex scenarios, and demonstrating outstanding performance, are hindered by complex structure and control, high costs, and high barriers to engineering application, and are thus suitable for complex underwater missions and extreme environment adaptation.

Overall, the swimming performance of robotic fish is strongly correlated with the type of variable-stiffness structure: smart material-driven systems are suitable for miniaturized, high-precision scenarios, bio-inspired driven systems excel in energy efficiency and biomimetic fidelity, fluid-driven systems are more appropriate for medium-to-large-sized robots requiring wide frequency adaptability, and hybrid-driven systems achieve balanced performance in complex scenarios through multi-mechanism synergy.

## 7. Conclusions

This article has presented a systematic review of variable stiffness methodologies in biomimetic robotic fish, categorizing state-of-the-art mechanisms into smart material-driven, bio-inspired, fluid-driven, and hybrid configurations. The comparative analysis elucidates the specific advantages and technical limitations inherent to each category, demonstrating that variable stiffness technology possesses significant potential to enhance the hydrodynamic performance and environmental adaptability of robotic fish.

Future research in smart material-driven variable stiffness must shift from basic material implementation to resolving fundamental thermodynamic and electromechanical bottlenecks. For thermally responsive materials like SMA, thermal hysteresis latency precludes high-frequency maneuvering. For electrically responsive materials like piezoelectric ceramics, research must focus on overcoming minuscule strain limits without relying on bulky mechanical linkages.

The future trajectory for bio-inspired variable stiffness structures depends on transitioning from mechanically complex, open-loop hardware to elegantly designed intelligent systems. The integration of DRL algorithms, such as Deep Deterministic Policy Gradient models trained in high-fidelity CFD simulations, is essential for allowing robotic fish to autonomously discover non-intuitive stiffness combinations that minimize the Cost of Transport in highly unstructured, turbulent conditions.

To elevate fluid-driven variable stiffness systems from shallow-water prototypes to autonomous deep-sea explorers, future research must comprehensively address extreme environmental resilience and decentralized actuation. The most critical frontier is eliminating compressible pneumatic systems entirely in favor of fully liquid-filled, pressure-compensated soft robotic architectures capable of operating in hadal zones.

Because hybrid-driven systems are inherently highly complex, future research must prioritize the development of unifying computational frameworks, multimodal sensor integration, and advanced AI co-design methodologies. The most pressing requirement is the establishment of comprehensive, multi-physics dynamic models that mathematically formulate the highly coupled, chaotic interactions between vastly different propulsion modes, such as the hydrodynamic interference between a classical vector thruster’s wake and a biomimetic undulating fin.

## Figures and Tables

**Table 1:** Swimming performance of different types of robotic fish.

Robotic Fish	Length (m)	Max. Speed (m/s)	Max. Speed (BL/s)	Min. Radius (BL)	Max. Turn Rate (°/s)	Strouhal Number
Soft robotic fish [70]	0.0043	0.05 (100 Hz)	11.63	NA	NA	NA
Octobot [83]	NA	0.025 (1.5 Hz)	0.5	NA	NA	NA
SoRoFAAM-1 [85]	0.28	0.087 (1.5 Hz)	0.31	0.15	18	0.61
Robotic fish with TSCP [69]	0.576	0.091(5 Hz)	0.16	NA	NA	0.295
Dual caudal fins robotic fish [87]	0.06	0.045(4 HZ)	0.75(4 Hz)	0.23	NA	NA
Robotic jellyfish [88]	0.1	0.01(0.8 Hz)	0.1	NA	NA	NA
Tuna-inspired platform [89]	0.35	0.7	2	NA	NA	NA
TenFiBot [71]	0.36	0.31	0.87	NA	NA	0.35–0.5
TenFiBot incorporating soft fish skin and tail [93]	0.36	0.512 (5 Hz)	1.423	NA	NA	0.3–0.55
Flexible fishtail with MFC [91]	NA	0.1235 (3 Hz)	NA	NA	NA	NA
Robotic fish with DEA [92]	0.262	0.0102 (2 Hz/3 Hz)	0.04	NA	NA	NA
Soft robotic jellyfish [95]	0.25	NA	NA	NA	NA	0.2159
Robotic fish [96]	0.53	0.43 (2.5 Hz)	0.81	NA	NA	NA
T-Fish [68]	0.45	0.47 (2.2 Hz)	1.04	0.31	153.3	NA
Robotic dolphin [97]	0.66	0.74 (2.88 Hz)	1.12	NA	NA	NA
Tensegrity robotic fish [98]	0.36	0.295 (2.75 Hz)	0.84	0.68	46.6	NA
On-VSFR [99]	0.41	0.35 (2.4 Hz)	0.86	NA	NA	0.45–0.65
Robotic tuna [100]	0.41	0.54 (2.4 Hz)	1.31	NA	NA	0.4–0.5
Fish-like robot [103]	0.24	0.5 (3 Hz)	2.1	0.9	NA	NA
Robotic fish [104]	0.4	0.43 (3 Hz)	1.07	NA	NA	NA
TenFiBot-HFS [101]	0.36	0.41 (5 Hz)	1.13	NA	NA	NA
Robotic fish with MAVSPJ [106]	0.3	0.31 (3 Hz)	1.03	NA	NA	0.58–0.79
TenFiBot-BIS [102]	0.36	0.4 (5 Hz)	1.1	NA	NA	NA
Flatfishbot [108]	0.2	0.103	0.515	NA	NA	NA
Robotic fish with FTFA [111]	NA	NA	≈6 (36.7 Hz)	NA	NA	0.355–0.38
Robotic fish [113]	0.53	0.052 (0.75 Hz)	0.1	NA	4.7 (0.75 Hz)	NA
HVS robotic fish [116]	0.615	0.389 (1.25 Hz)	0.63	0.14	94.1	NA
Soft robotic fishtail [117]	NA	0.18 (0.66 Hz)	NA	NA	NA	NA
HasorTuna [114]	0.576	0.48 (4 Hz)	0.84	3.3	NA	0.6–1.1
HyperTuna [115]	0.52	0.58 (6 Hz)	1.12	0.47	NA	NA
Robotic fish [118]	0.18	0.0288 (0.625 Hz)	0.16	NA	NA	NA

**Table 2:** The average value of the maximum swimming speed for each type of robotic fish.

Variable Stiffness Structures	Smart Material Type	Bio-Inspired Type	Fluid-Driven Type	Hybrid-Driven Type
Average Max. Speed (BL/s)	0.364	1.116	0.641	0.16

**Table 3:** Advantages, disadvantages and application scenarios of various types of variable stiffness structures.

Variable Stiffness Structure		Advantages	Disadvantages	Application Scenarios
Intelligent Material Type	Thermal Response Type	Stable material performance Reversible stiffness switching Miniaturized design and compact structure	Difficult temperature control Slow response speed Unverified performance stability	Miniature underwater monitoring Narrow-space operations Low-speed stable propulsion
	Electrical Response Type	High propulsion efficiency Suitable for narrow space operations	Small output displacement High driving voltage requirement Narrow stiffness adjustment range	Micro robotic fish for precision tasks Confined underwater environments High-frequency micro-maneuvering
Bio-inspired Type	Antagonistic Control Type	Suitable for multi-task scenarios Significant efficiency optimization High bionic fidelity	Energy loss Complex control Structure dependent on drive	High-frequency swimming robots Marine environment detection Efficient long-duration propulsion
	Structural Control Type	Strong material compatibility Lightweight structure	Limited mobility Insufficient high-frequency performance High processing difficulty	Specific flow regime operations Low-energy consumption tasks Customized shape-adaptive propulsion
	Mechanical Control Type	Simple structure and low cost Low energy consumption Suitable for multi-joint integration	Narrow stiffness adjustment range Prone to fatigue after long-term use	Medium-low speed stable swimming Long-endurance underwater monitoring Multi-joint bionic robots
Fluid-driven Type	Pneumatic Type	Fast response speed Wide stiffness range Lightweight structure	Dependence on external equipment High energy consumption and short endurance High sealing requirements	Nearshore monitoring Short-duration underwater operations Low-speed gliding and load transportation
	Hydraulic Type	Fast response speed Accurate and wide stiffness adjustment range Strong load-bearing capacity	Complex system Energy waste High maintenance difficulty	High-performance robotic fish Deep-sea professional operations High-precision maneuvering and heavy-load tasks
Composite-driven Type		Significant performance optimization Suitable for complex underwater scenarios Function integration	Complex structure/control Superimposed energy consumption High integration difficulty	Complex underwater missions Extreme environment adaptation Multi-performance requirement tasks

## Data Availability

Data are available upon reasonable request from the corresponding author.
